# Comparison of Expandable and Locked Intramedullary Nailing for Humeral Shaft Fractures

**DOI:** 10.7759/cureus.18833

**Published:** 2021-10-17

**Authors:** Mehmet Akdemir, Çağdaş Biçen, Mustafa Özkan, Ahmet Ekin

**Affiliations:** 1 Orthopedics, Özel Izmir Ekol Hospital, Izmir, TUR; 2 Orthopedics and Traumatology, Izmir University of Economics, Medical Park Hospital, Izmir, TUR; 3 Orthopedics and Traumatology, Dokuz Eylül University Hospital, Izmir, TUR

**Keywords:** humerus shaft, fracture, intramedullary nail, expandable nail, trauma

## Abstract

Introduction

In this study, we clinically and radiologically investigated whether the application of expandable nails for surgical treatment of humeral shaft fractures has an advantage over locked intramedullary nails.

Methods

Patients treated with intramedullary fixation due to humeral shaft fractures in our clinic were investigated retrospectively. Patients with fractures of type 12A and 12B according to the AO classification in the middle 1/3 shaft region of the humerus were divided into two groups as those receiving fixation with expandable nails and with locked intramedullary nails. The union rate, union time, Q-DASH scores, duration of surgery, and complication rates were statistically compared between the two groups.

Results

The study included 38 patients with clinical follow-up from among 47 patients; 20 patients received fixation with locked intramedullary nails and 18 with expandable nails. The mean age of the patients was 56.92 (19-91) years and 53% (n=20) were men while 47% (n=18) were women. During statistical evaluation, a statistically significant difference was found between the groups for union (100% and 72.2%) and complication rates (6% and 13%). More union and lower complication rates were found in patients treated with locked intramedullary nails. In comparing the mean of surgical times (71.1 and 30.2 min), expandable nails had a shorter surgical time. However, there was no statistically significant difference between the union time and Q-DASH scores between the two groups.

Conclusion

Locked intramedullary nails are a better fixation method than expandable nails due to the low complication rate and high rate of union. However, due to shorter surgery time, expandable nailing is an alternative method in limited cases.

## Introduction

Although humeral diaphyseal fractures can be treated with good results by conservative methods, the tendency toward more surgical treatments is increasing due to the recent increase in patient expectations [1,2]. Conservative treatment consists of cast or brace applications and may be prolonged to six months, decreasing the satisfaction of the patients as it often creates shoulder and elbow movement limitations that require long-term physical therapy programs to be resolved [3].

In the surgical treatment of humeral diaphyseal fractures, the options include intramedullary nails, plates, and external fixators. Although successful results are obtained with the plate fixation method, it has disadvantages such as wide soft tissue dissection, development of avascularity in the fracture ends, blood loss, and long surgical time [4]. Although external fixator methods are an alternative treatment of humeral diaphyseal fractures, they are generally only preferred in cases such as open fractures and polytrauma due to patients’ inability to tolerate surgery of longer durations [5].

Intramedullary nailing has become a preferable method for reasons such as being minimally invasive, causing less damage to soft tissues and less blood loss, and having a shorter surgical time [6,7]. With locked intramedullary nailing, the surgical time is extended during distal locking, additional fluoroscopy is required, and there is a risk of radial nerve damage. In order to avoid those disadvantages, the expandable nail system was developed [8,9].

In this study, we retrospectively compare the clinical and radiological results of patients with similar demographic and fracture features for whom we applied locked intramedullary nails or expandable nails due to humeral diaphyseal fractures.

## Materials and methods

All procedures followed were in accordance with the ethical standards of the responsible institutional committee on human experimentation and the revised 1975 Declaration of Helsinki. Patients operated on in our clinic for humeral diaphyseal fractures with intramedullary nailing were evaluated retrospectively. The humeral diaphysis was defined as the area between the pectoralis major attachment site proximally and the supra-epicondylar region distally. The inclusion criteria included having been operated on with intramedullary nailing due to acute humeral fracture of type 12A or 12B according to the AO classification in the middle one-third shaft region and being followed for at least 12 months after surgery. Gustilo-Anderson type 3 open fractures, patients under 18 years of age, pathological fractures, fractures with intra-articular extension, and type 12C fractures were excluded.

Demographic data, perioperative and postoperative findings, and clinical results were obtained from the medical records of the hospital and from the patients’ files. Clinical evaluation was performed with Q-DASH scoring in the patients’ last follow-up visits. Preoperative, postoperative, and control radiographs were obtained from the hospital picture archiving and communication system (PACS) system. Union of the fracture was accepted when cortical bridging and callus formation were observed in at least three cortices in direct radiographs. Osteolysis at the fracture ends, implant failure, osteolysis around the implant, and insufficient bone formation in the fracture line after six months were evaluated as radiological nonunion. Complications regarding radial nerve injury, implant failure, fracture, and development of infection were recorded. There were both low-energy and high-energy traumas in our patient group. Fractures that occurred after a simple fall were considered as low-energy, while fractures that occurred due to reasons such as falling from a height or traffic accidents were considered as high-energy traumas.

The patients were divided into two groups as those treated with locked intramedullary nails and those treated with expandable nails. Patients’ treatment methods were decided based on the surgeon’s choice. The patients received intramedullary nails with both antegrade and retrograde methods. Which method to use was decided based on the patient’s symptomatic shoulder pain. Retrograde administration was preferred for patients with shoulder pain or degeneration in direct radiographs before fracture formation.

**Figure 1:**
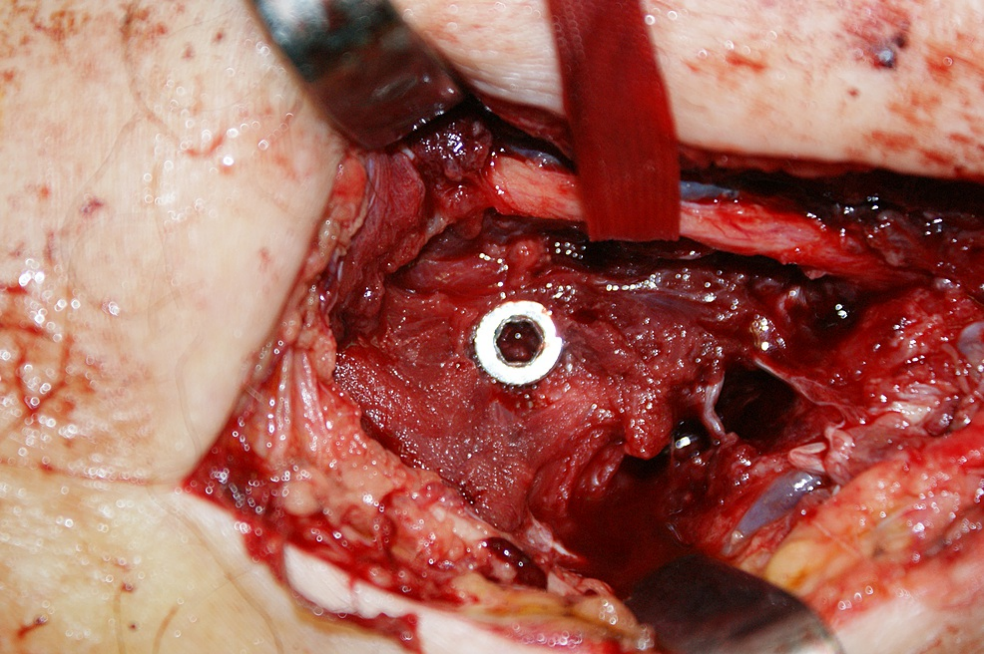
Due to this proximity, the radial nerve should be protected during distal locking.

In the antegrade approach, with a proximal lateral incision in the beach chair position, the rotator cuff was separated and the entrance hole was opened with the help of an awl under the guidance of the fluoroscopy device. In cases where closed reduction was not fully achieved, the fracture line was opened with a small incision and reduction was then achieved. After the nail was inserted, the proximal and the distal parts of the locked intramedullary nail were locked with at least one screw. While applying the distal lock screw, a small skin incision was performed and the radial nerve was protected (Figure 1). After the procedure, the rotator cuff was repaired. With a retrograde approach, a 5 cm longitudinal incision was made proximally to the posterior of the elbow joint in the prone position. The triceps tendon split was separated and the entrance hole was opened with the help of an awl by fluoroscopy guidance over the olecranon fossa. Afterwards, the nail inlet was gradually extended to allow the entrance of the nail. The fracture was reduced and then the nail was placed. As in the antegrade application, locking was done with at least one distal proximal and one distal screw. Again, during distal locking, the radial nerve was dissected and protected. Patients who received expandable nails were treated with the Fixion IM Nail © (Disc-O-Tech, Herzliya, Israel). After applying the nail in an antegrade or retrograde manner, the nail was expanded with the help of a directional valve delivering saline solution. After reaching 70 bar pressure and maximum width, the nail was fixed. After fluoroscopy control, the layers were closed. No reaming was applied in the application of locked nails or expandable nails. Nail thickness was determined according to preoperative and perioperative evaluations.

All patients were operated on under general anesthesia. Preoperatively, 1 g of cefazolin sodium was administered intravenously, two more doses were given after the operation, and oral cefuroxime at 500 mg was continued for seven days in two doses. Low-molecular-weight heparin (enoxaparin sodium) in a single dose of 0.4 mL was applied postoperatively for deep vein thrombosis prophylaxis. It was continued together with acetylsalicylic acid at 100 mg for 10 days after the operation. After the operation, the patients were followed with a shoulder arm strap. Shoulder and elbow movements started from the first postoperative day and patients were encouraged to use the arm.

Patient data were uploaded to Microsoft Excel. Statistical analysis was performed with the help of SPSS Statistics v. 17 (SPSS Inc., Chicago, IL). The patients were divided into two groups of those treated with locked intramedullary nails and those treated with expandable nails. The comparison of numerical data (age, Q-DASH score, union time, follow-up time, duration of surgery) between the two groups was done with the Mann-Whitney U test. Comparison of categorical data (gender, side, presence of union, complication rate, type of fracture) was done with Pearson’s chi-square test or Fisher’s exact test. If there were more than five values in the cross-table, Pearson’s chi-square test was applied, and if there were fewer than five in the table, Fisher’s exact test was applied. Statistical significance was accepted at a 95% confidence interval and p < 0.05. When p < 0.05, it was accepted that there was a statistically significant difference between the two groups.

## Results

It was found that 47 patients were treated with intramedullary nails for humeral diaphyseal fracture of type 12A or 12B in our clinic within the considered period of time. Nine of those patients had insufficient follow-up data and 38 patients were thus included in the study. The mean age of these patients was 56.92 (19-91) years and 53% of the patients were male while 47% were female. Furthermore, 55% of the fractures were of the right and 45% of the left humerus. Eighteen patients were operated on with expandable nails and 20 patients were operated on with locked nails. The age, gender, and side distributions were similar between the two groups (p=0.704, p=0.758, p=0.973; p > 0.05). Of the 18 patients with expandable nails, seven were treated with a retrograde and 11 with an antegrade entry site. Among the patients with locked intramedullary nails, only one was treated via a retrograde access site.

According to the AO fracture classification, 31 patients had type 12A and 7 patients had 12B fractures (Table 1). There was no statistically significant difference found in fracture types between the groups (p=0.410; p > 0.05). Comparing the severity of the trauma of the patients, 45% had low-energy and 55% had high-energy trauma; there was again no statistically significant difference between the groups in this regard (p=0.321; p > 0.05). Ten (26%) of the patients had additional limb injuries [pelvis (1), hip (1), femur (2), tibia shaft (2), distal tibia (1), clavicle (1), olecranon (1), distal radius (1)]. Four (11%) patients had open fractures. Two patients had type 1 and two patients had type 2 open fractures according to the Gustilo-Anderson classification. The groups were statistically similar in terms of additional injuries and open fractures (p=0.278, p=1.000; p > 0.05) (Table 2).

**Table 1:** Fracture characteristics of patients according to AO classification. Exp. nail: expandable nail; LIN: locked intramedullary nail

Fracture classification	Total		Exp. nail.		LIN	
12A1	3	31 (81.6%)	2	16 (88.9%)	1	15 (75%)
12A2	3		3		0	
12A3	25		11		14	
12B1	4	7 (18.4%)	1	2 (11.1%)	3	5 (25%)
12B2	3		1		2	
12B3	0		0		0	
12C1,2,3	0	0	0	0	0	0

**Table 2:** General demographic characteristics of the patients. *; Mann Whitney U test, **; Pearson’s chi-square test, ***; Fisher’s exact test.

Demographics		General	Expandable nail	Locked intramedullary nail	p value
Age (years)		56.92 (19-91)	57.67	56.25	0.704*
Gender	Male	20 (53%)	9 (50%)	11 (55%)	0.758**
	Female	18 (47%)	9 (50%)	9 (45%)	
Side	Right	21 (55%)	10 (56%)	11 (55%)	0.973**
	Left	17 (45%)	8 (44%)	9 (45%)	
Fracture type	Low energy	17 (45%)	7 (39%)	10 (50%)	0.321**
	High energy	21 (55%)	11 (61%)	10 (50%)	
Additional injury		10 (26%)	3 (17%)	7 (35%)	0.278***
Open fracture		4 (11%)	2 (11%)	2 (10%)	1.000***

When the clinical and radiological results of the patients were compared, there was no statistically significant difference between the groups in terms of Q-DASH scores, union times, or follow-up times (respectively p=0.410, p=0.092, p=0.918; p > 0.05). However, it was observed that the union rate was statistically significantly higher in the locked nail group (p=0.017; p < 0.05). A statistically significant difference was also found upon comparing the mean surgery times of the groups (p < 0.000; p < 0.05). The average duration of surgery in the expandable nail group was 30.2 minutes, while in the locked intramedullary nail group it was 71.1 minutes (Table 3).

**Table 3:** Distribution and comparison of clinical and radiological results in the general patient group and the expandable nails and locked intramedullary nails groups *; Mann Whitney U test, **; Fisher’s exact test

Clinical and radiologic results	General	Expandable nail	Locked intramedullary nail	p value
Q-DASH score	18.47 (0-97)	25.22	12.40	0.410*
Union time (months)	4.85	5.69	4.25	0.092*
Union rate	86.8%	72.2%	100%	0.017**
Follow up time (months)	26.4	25.8	26.9	0.918*
Surgical time (minutes)	51.7	30.2	71.1	0.000*

Shoulder problems developed in five patients, distal humeral fracture in four, infection in one, and radial nerve injury in four. Nonunion occurred in five cases. Shoulder problems regressed in two patients after physical therapy, but the nail had to be removed after union in the other three cases. The patients who developed distal humeral fracture underwent plate fixation. Spontaneous improvement occurred within six months in the patients with radial nerve injury. A patient with an open fracture developed an infection; improvement was achieved with wound debridement and oral antibiotic treatment without the need for nail removal. The nails of the five patients with nonunion were removed and they were treated with autogenous grafting and plate fixation. A statistically significant difference was found upon comparing the complication rates of the groups (p=0.009) (Table 4).

**Table 4:** Complications *; Pearson’s chi-square test N/A: not available

Complications	General	Expandable nail	Locked intramedullary nail	p value
Shoulder pain	5 (13.2%)	2 (11.1%)	3 (15.0%)	0.009*
Distal humerus fracture	4 (10.5%)	3 (16.7%)	1 (5.0%)	
Radial nerve neuropraxis	4 (10.5%)	2 (11.1%)	2 (10.0%)	
Infection	1 (2.6%)	1 (5.6%)	N/A	
Nonunion	5 (13.2%)	5 (27.8%)	N/A	
Total	19 (50.0%)	13 (72.2%)	6 (30%)	

## Discussion

In our study, we wanted to examine the clinical and radiological results after locking and expandable nail applications in cases of humeral diaphyseal fractures. We also wanted to explore the differences between antegrade and retrograde approaches in both groups.

**Figure 2:**
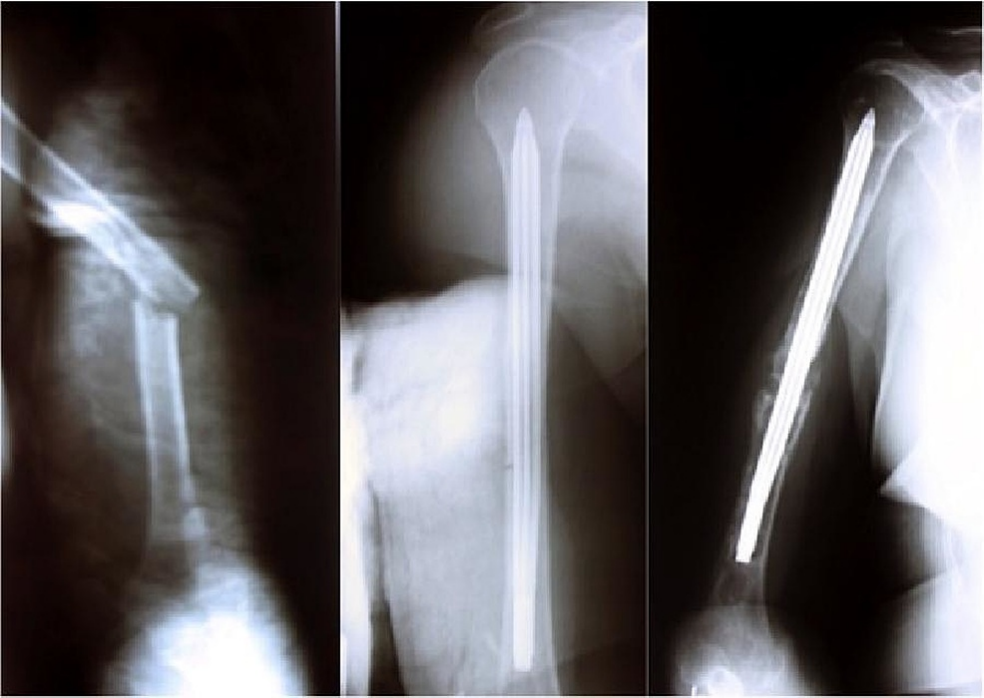
Fixation of humeral shaft fracture with retrograde expandable nail. Complete reduction was obtained in early postop radiography. However, osteolysis and nonunion developed in the distal part according to follow-up radiographs.

In the treatment of humeral diaphyseal fractures, intramedullary nails are preferred because they can be applied less invasively. Locked nails are designed as an alternative to nonlocked intramedullary nails, as nonunion rates are high due to weak rotational stability. However, with locked nails, problems such as radial nerve injury, surgical difficulty, and prolonged operation time have occurred due to distal locking. Expandable nails were developed to eliminate the problems encountered in distal locking [6,10] (Figure 1). The purpose of expandable nails is to ensure the stable fixation of the humeral diaphysis and eliminate the necessity of distal locking [8]. In biomechanical studies, it has been reported that locked intramedullary nails provide better rotational stability than expandable nails [11,12]. One of the main theoretical advantages of the expandable nail over locked nails is the ability to achieve an interference fit within the medullary cavity without reaming (Figure 2) [11,13].

**Figure 3:**
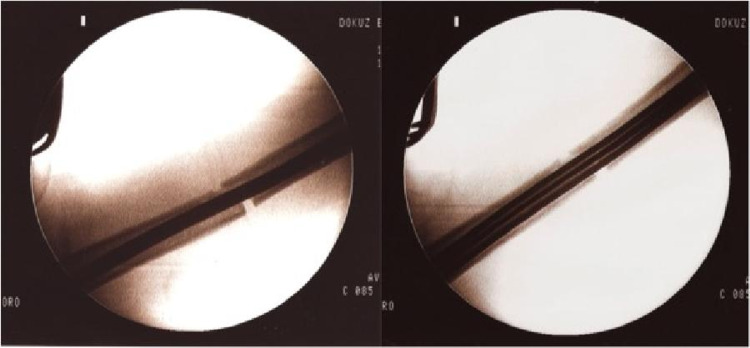
Fluoroscopic images during the application of the expandable nail. After the nail is inflated, distraction appears in the fracture line.

**Figure 4:**
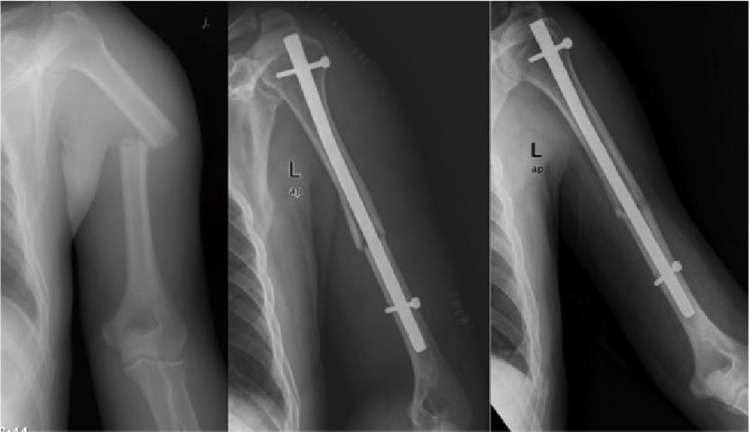
Union is achieved after application of locked intramedullary nail.

It is recommended to use expandable nails in the humeral diaphysis in the middle one-third and for transverse or rotational stable fractures [14]. Although good clinical results have been reported with expandable nails when applied in this fracture group, there are also studies reporting that expandable nails have failed and that the risks of complications and secondary operations are high [9,15-21]. Loss of rotational stability, osteolysis and nail migration, and deflation of the expandable nail (i.e. loss of the expandability feature) were identified as causes of failure [9, 15-21]. In a review study, the nonunion rate of expandable nails in humeral fractures was found to be 7.8% [8]. Our nonunion rate was 27.8% and all patients who had nonunion were in the expandable nail group (Figures 3-4). Union could be achieved in all patients treated with interlocking nails. The nonunion rate was statistically significantly higher with expandable nails compared to interlocking nails (p=0.017; p < 0.05). We thought that this high rate was related to insufficient rotational stability. However, the Q-DASH scores (mean: 25.22) and mean union time (5.69 months) of patients treated with expandable nails who achieved union did not differ statistically (p=0.410, p=0.092; p > 0.05). In the expandable nail group, there were statistically significantly more complications compared to the locked nail group (p=0.009; p < 0.05). Our surgical times for expandable nail application were statistically significantly shorter than those for locked intramedullary nails (p=0.000; p > 0.05).

After antegrade intramedullary nailing, shoulder joint problems can be seen in up to 30% of cases [22], and nailing with the retrograde method has been proposed to avoid shoulder problems [23]. We also had shoulder problems in five (13.2%) patients for whom we had applied antegrade nails. Two of them were treated with expandable nails and three were treated with locked intramedullary nails. Physical therapy resolved the complaints for two patients, but for three patients implant removal after bone union had to be performed.

With retrograde nailing, problems related to elbow circumference can be encountered; technically, it is difficult to expand the entrance of the distal humerus [11]. During reaming, the possibility of disintegration around the fracture and the probability of supracondylar fracture increase. In a biomechanical study, the probability of a fracture in the distal humerus was found to increase statistically due to the olecranon fossa entry nail or metaphyseal entry nail [11]. Supracondylar fractures occurred in four of seven patients for whom we applied retrograde nails. One of these occurred intraoperatively and three occurred in the first three months after the operation. Three of these four patients were treated with expandable nails. This was due to biomechanical weakening; although the humeral anatomy has an anterior opening distally, this can be explained by the fact that the nail is flat and the supracondylar region is weakened due to the increase of intramedullary pressure during expansion [24, 25]. Although there are contradictory studies on this issue [16], we do not recommend the retrograde application of expandable nails in line with our results.

In humeral fractures, radial nerve problems are seen in about 11% of cases [26]. In antegrade nailing, and especially during distal locking from the lateral to the medial, radial nerve damage can occur if careful attention is not paid [27]. For this reason, while distal locking is being performed in locked intramedullary nailing, it is recommended to perform the locking while seeing the radial nerve through a larger incision. This increases the surgical time but also reduces the rate of radial nerve injuries. Radial nerve injury developed in four of our patients (10.5%). Two of them were in the locked nail group and two were in the expandable nail group. Radial nerve findings regressed spontaneously within six months for all patients. In our study, we found that the expandable nail group had no advantage in terms of radial nerve complications.

Our study has some limitations. First of all, our study has a retrospective design. Secondly, we had a limited number of patients in this study. Thirdly, the follow-up of the patients was relatively short. Prospective studies on this subject with higher numbers of patients will yield more meaningful results. To our knowledge, our study is the first in the literature comparing the clinical results of locked intramedullary nails and expandable nails in humeral shaft fractures.

## Conclusions

Locked nails are superior in humeral diaphyseal fractures compared to expandable nails with lower complication rates and higher rates of union. However, expandable nails should still be considered a better alternative to locked intramedullary nails for patients for whom prolonged surgical time will be more risky. In our study, the surgical time of the expandable nail application was significantly shorter than that of the locked intramedullary nail. Expandable nails, in our opinion, may be used as an alternative in cases in which it is essential to keep the surgical time short, such as in cases of osteoporosis or polytrauma and patients with severe comorbidities. In patients other than this limited group, we recommend the application of locked nails with intramedullary fixation for humeral diaphyseal fractures.
